# Choriocapillaris flow impairment surrounding geographic atrophy correlates with disease progression

**DOI:** 10.1371/journal.pone.0212563

**Published:** 2019-02-22

**Authors:** Marco Nassisi, Elmira Baghdasaryan, Enrico Borrelli, Michael Ip, Srinivas R. Sadda

**Affiliations:** 1 Doheny Image Reading Center, Doheny Eye Institute, Los Angeles, California, United States of America; 2 Department of Ophthalmology, David Geffen School of Medicine at UCLA, Los Angeles, California, United States of America; 3 Ophthalmology Clinic, Department of Medicine and Science of Ageing, University G. D'Annunzio Chieti-Pescara, Chieti, Italy; Bascom Palmer Eye Institute, UNITED STATES

## Abstract

**Purpose:**

To evaluate the correlation between the choriocapillaris (CC) flow alterations around geographic atrophy (GA) and the GA yearly growth rate (yGR) in patients with dry age-related macular degeneration (AMD).

**Methods:**

We retrospectively reviewed and analyzed spectral domain optical coherence tomography (SD-OCT) and SD-OCT angiography images of consecutive patients with GA acquired using the Cirrus OCT at the Doheny Eye Centers between 2015 and 2017. All eligible patients had one 6 x 6 mm OCTA scan acquired during the first visit (considered as baseline) and two fovea-centered 512 x 128 macular cubes (6 x 6 mm) acquired at baseline and after a minimum of 12 months.

**Main outcome measures:**

The fundus images from the OCT volumes were used to manually delineate the GA area and calculate the yGR after square root transformation. The *en-face* angiogram at the level of the CC was analyzed for the percentage of flow voids (FV) outside the atrophic lesion (FV_OUT_) and in the *para-* and *peri-atrophy regions* (FV_500_ and FV_1000_ respectively; two concentric 500 μm wide rings around the atrophy edge). These values, together with the difference between FV_500_ and FV_1000_ (ΔFV), were then correlated with the corresponding yGR.

**Results:**

Thirty-three eyes of 23 patients were eligible for the analysis. The mean yGR was 0.23 ± 0.17 mm/years.

At baseline, the mean FV_OUT_ was 41.86 ± 2.71%, while FV_500_ and FV_1000_ were 46.4 ± 4.17% and 42.51 ± 2.65% respectively. The mean ΔFV was 3.89 ± 2.6%. While in the univariable analysis, the yGR was significantly associated with FV_500_ and with ΔFV (both p < 0.001), in multivariable model the association remained significant only with ΔFV (p < 0.001).

**Conclusions:**

Our study reports a correlation between the CC flow impairment around the atrophic lesions and their yGR in patients with GA. If replicated in future longitudinal studies, the choriocapillaris FV in the *para-and peri-atrophy regions* may prove to be useful parameters for evaluating the prognosis of these eyes.

## Introduction

Geographic atrophy (GA) is a late stage manifestation of dry age-related macular degeneration (AMD). The classic definition of GA is based on the observation on 30 or 35 degrees color fundus images of sharply delineated roughly round or oval regions of hypopigmentation or depigmentation with increased visibility of the underlying choroidal vessels measuring at least 175 μm in diameter[1].

A recent optical coherence tomography (OCT) based classification from the Classification of Atrophy Meeting (CAM) defines GA as a complete retinal pigment epithelium (RPE) and outer retinal atrophy (cRORA), in the absence of choroidal neovascularization (CNV) (present or previous)[2]. Criteria for identifying atrophy on OCT as defined by CAM include a: (1) region of hypertransmission of at least 250 μm in diameter in any lateral dimension, (2) zone of attenuation or disruption of the RPE of at least 250 μm in diameter, and (3) evidence of overlying photoreceptor degeneration[2].

At present, however, definitions of GA do not specifically take into account the choriocapillaris (CC), though it is well known that GA patients have significant CC disruption under the area of the lesion as demonstrated by multiple histologic studies[3–6]. Research investigations of the CC have increased dramatically over the last several years in large part due to the development and availability of OCT angiography (OCTA). OCTA allows the *in vivo* visualization of the retinal and inner choroidal microcirculation in a depth-resolved fashion without the need for invasive dye injection[7]. While histologic studies provide a direct visualization of the vascular structure, OCTA imaging is based on the presence of motion or blood flow, and therefore the absence of OCTA signal means the absence of blood flow above the threshold of detection rather than necessarily a complete absence of blood flow[8]. OCTA studies of eyes with GA, have demonstrated *in vivo* impairment of the CC flow under the atrophic patches[9] and even under the areas surrounding the atrophic lesions where the RPE appears to be still intact[10]. In particular *Sacconi et al*. recently compared the CC flow under the areas rated as hyperautofluorescent on fundus autofluorescence (FAF) images surrounding the GA with similar areas rated as isoautofluorescent and demonstrated that even though the CC flow impairment is present in both areas, it is significantly higher in the hyperautofluorescent areas which are thought to represent regions of impaired RPE cells. Based on this observation, they hypothesized that the first injury in GA occurs at the level of the CC. If one accepts this theory, one might further hypothesize that impairment of CC flow around the GA lesion may influence the subsequeny enlargement of these lesions. Thus, the aim of our study is to investigate the correlation between the impairment of the CC flow surrounding the atrophic lesions and the yearly growth rate (yGR) of GA.

## Methods

In this retrospective study, we collected and analyzed spectral domain (SD)-OCT and SD-OCTA images of consecutive patients with GA acquired at the Doheny Eye Centers between 2015 and 2017 using Cirrus HD-OCT (Carl Zeiss Meditec, Dublin, CA).

Eligible patients had GA on OCT (as defined by CAM[2]) in at least one eye and no evidence of any other pathology involving the macula. Eyes with non-visually significant vitreoretinal interface disease, such as a subtle epiretinal membrane only visible by OCT, were not excluded. All eligible patients needed to have one 6 x 6 mm OCTA scan acquired during the first visit (considered as baseline) and two fovea-centered 512 x 128 cubes (6 x 6 mm) acquired at baseline and at a second visit with a follow-up of at least 12 months. Only subjects with scans that fulfilled the image quality acceptance criteria (signal strength >7, absence of motion artifact) of the Doheny Image Reading Center (DIRC) according to the evaluation of two certified readers, were selected and analyzed[11,12].

Data collection and all analyses for this study were approved by the institutional review board (IRB) of the University of California–Los Angeles (UCLA). The study was performed in accordance with the Health Insurance Portability and Accountability Act and adhered the principles of the Declaration of Helsinki. A waiver of informed consent was granted by the IRB for this retrospective analysis.

### GA progression rate

The two fovea-centered 512 x 128 macular cubes (6 x 6 mm) for each eligible eye were analyzed in order to calculate the yGR of the GA. In accordance with the CAM criteria, GA was identified based on the presence of contiguous hypertransmission (primarily in the choroid) > 250 microns, with overlying evidence of an RPE defect of >250 microns as well as thinning of the outer retina.

On both images two independent graders (MN and EB) manually delineated the GA region using the *en-face* OCT fundus image as previously described[13,14], using ImageJ software version 1.50 (National Institutes of Health, Bethesda, MD; available at http://rsb.info.nih.gov/ij/index.html) [15]. This image is the *en face* reconstruction of the sum of all the signals coming from each of the acquired A-scans[16]. GA appears as a bright area on the image due to the increased penetration of light into the choroid caused by the overlying RPE and outer retina atrophy[16,17]. The borders of the GA region were manually segmented and verified using the corresponding structural B-scans to ensure the integrity of the RPE outside the selected area.

The GA areas were converted using the square root transformation in order to eliminate dependence of growth rates on baseline lesion measurements[18]. Then the yGR was measured as follows:

$$yGR=\frac{\sqrt{{GA}_{II}}-\sqrt{{GA}_{I}}}{FU}$$

where the GA_I_ and GA_II_ represent the atrophic areas at the first and second visit respectively expressed in square millimiters (mm^2^), while FU represents the follow-up time expressed in years. Eyes with atrophic areas extending beyond the 6 x 6 mm scans (at either visit) were excluded from the analysis, as it was impossible to establish an accurate growth rate in such cases.

### OCTA analysis

The 6 x 6 mm OCTA scan consisted of a 350 A-scans x 350 B-scan pattern. A fully-automated retinal layer segmentation algorithm was applied to the three-dimensional structural OCT data, in order to segment the CC slab as defined previously (10 μm thick starting 31 μm posterior to the RPE reference).[19] This segmentation was then applied to OCTA flow intensity data to obtain vascular images. Maximum projection analyses of the flow intensity were performed to generate the *en-face* images of the CC plexus (1024x1024 pixels). These images were analyzed using ImageJ software[15].

**Fig 1:**
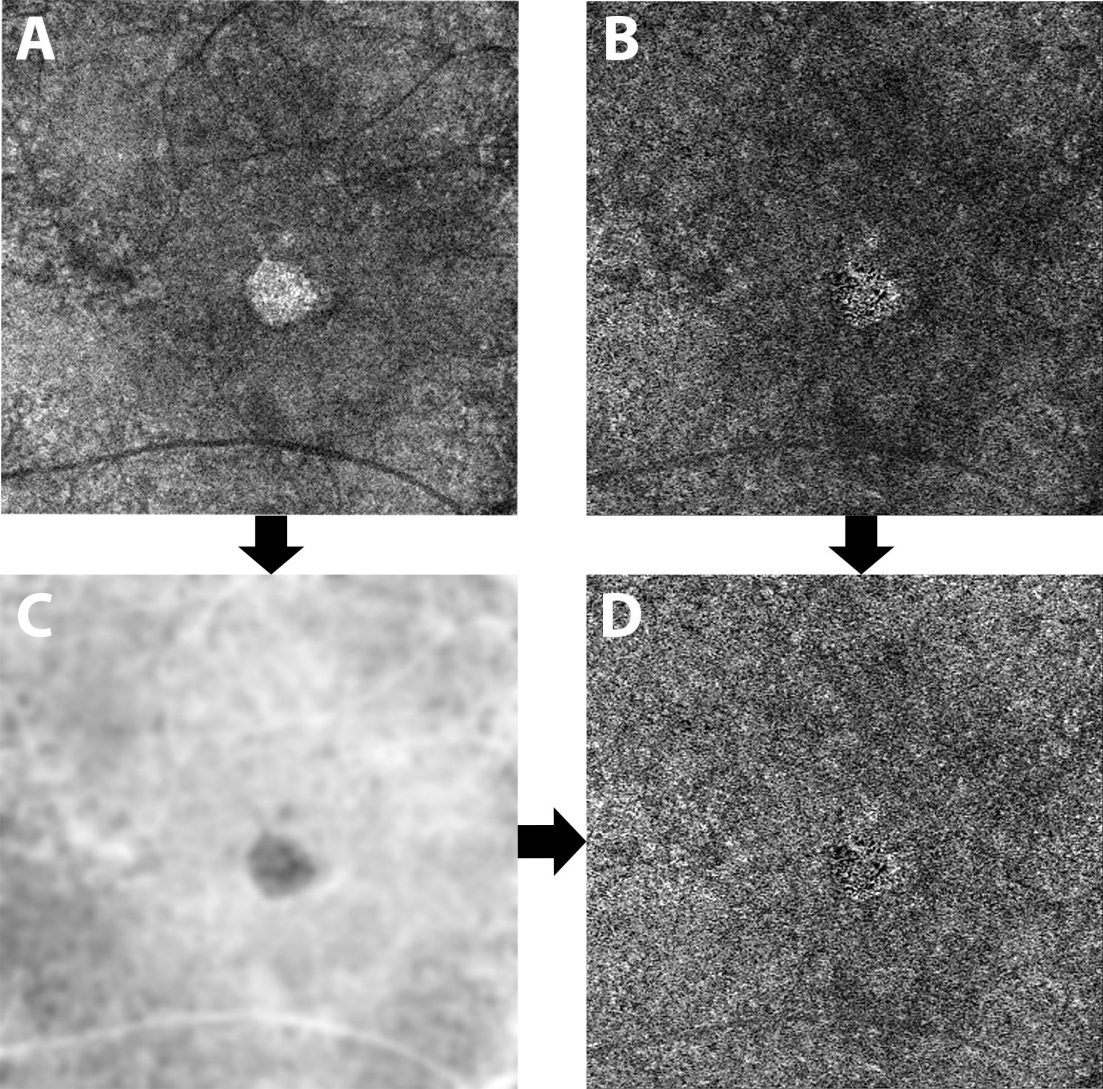
Compensation of the choriocapillaris signal. A 10 μm thick slab between 31 and 41 μm under the RPE reference was segmented to extract an *en-face* image of the choriocapillaris from the structural OCT (A) and the angiogram (B). After being inverted and blurred (C), the structure image was multiplied with the angiogram (B) in order to compensate for signal loss under areas of RPE alterations (i.e. drusen). The resulting image (D) was then analyzed.

To compensate for CC signal attenuation resulting from structural changes in the RPE/BM complex, a previously described method was applied [20,21]. Briefly, for each eye, a CC layer was first segmented from the structural OCT and the associated flow slab was then identified from the angiogram. An inverse transformation was applied to the *en-face* structural CC image to enhance the attenuated signal under drusen, where a Gaussian smoothing filter (3 X 3 pixel kernel) was used to minimize speckle noise. Then, a multiplication between the *en-face* CC flow image and the smoothed, inverted CC structural image was performed. In this way the shadowing effect under the drusen was compensated while the signal in the normal region remained the same (Fig 1).

The resulting compensated CC *en-face* image was then binarized for quantitative analysis of the signal voids using the Phansalkar method (radius, 15 pixels) as previously described[19,22,23].

**Fig 2:**
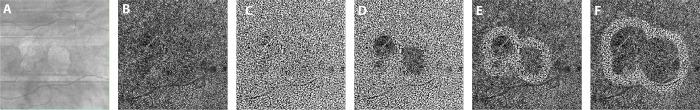
Protocol of image analysis. The OCT fundus image (A) was used to assist manual delineation of the geographic atrophy (GA) region. The compensated choriocapillaris angiogram (B) was thresholded in order to obtain a binarized picture where the black pixels represent the signal voids in the choriocapillaris flow (C). The percentage of flow voids was calculated outside the atrophy (D), within two concentric 500 μm wide regions surrounding the GA (*para-atrophy region (E)* and *peri-atrophy region (F*).

The quantitative analysis was performed outside the atrophic lesion and within two concentric 500-μm-wide rings immediately surrounding the GA lesion edge. The inner ring was defined as the *para-atrophy region* while the outer was defined as the *peri-atrophy region* (Fig 2).

The GA region was manually delineated using the OCT fundus image as previously described [13,24]. Then, the distance from the fovea (manually determined using the OCT angiogram of the superficial vascular plexus) was calculated from the centroid of the lesion. In case of multifocal lesions, the mean distance calculated from each focus was considered.

The area outside the GA region was processed with the ‘Analyze Particles’ command, in order to count the flow voids as a percentage of the area (FV_OUT_).

To isolate the *para-atrophy region*, we used the “Distance Map” ImageJ function on the GA region selection. Specifically, the “Distance Map” function provided delineation of a border 500 μm displaced from the atrophy edge. Furthermore, in case of multifocal lesions the “Distance Map” function on the “binarized” image allowed us to delimit those areas within 500 μm of the edge of all the atrophy lesions in the image (without any size limit), by excluding areas occupied by adjacent lesions. The *peri-atrophy region* was determined by applying the same method; specifically, the ‘distance map’ function was used to delineate a border displaced 500 μm from the *para-atrophy area* edge. The two areas were then separately processed with the ‘Analyze Particles’ command, in order to count the flow voids inside the *para-atrophy* (FV_500_) and *peri-atrophy regions* (FV_1000_). Then, the difference in percentage of flow voids between the two analyzed areas was calculated as:

$$\Delta FV=({FV}_{500}-{FV}_{1000})$$

The entire procedure was repeated by two independent, experienced operators (MN and EB) in order to investigate the repeatability of all measurements. All values were then averaged to perform the statistical analysis.

### Statistics

Statistical analyses were performed using SPSS Statistics version 20 (IBM, Armonk, NY). Intraclass correlation coefficients (ICC) were calculated for atrophy area, mean distance of the lesion from the fovea, and all flow voids measurements.

Pearson’s correlation coefficient (R) was calculated between all flow voids measurements and atrophy progression. A univariable analysis was performed with generalized estimating equations (GEE) to estimate the correlation between yGR (considered as dependent variable) and FV_OUT_, FV_500_, ΔFV, age, baseline GA area[25] and mean distance from the fovea [26]. Significant variants (if any) were included in the multivariable model.

In all analyses, P values < 0.05 were considered as statistically significant.

## Results

**Fig 3:**
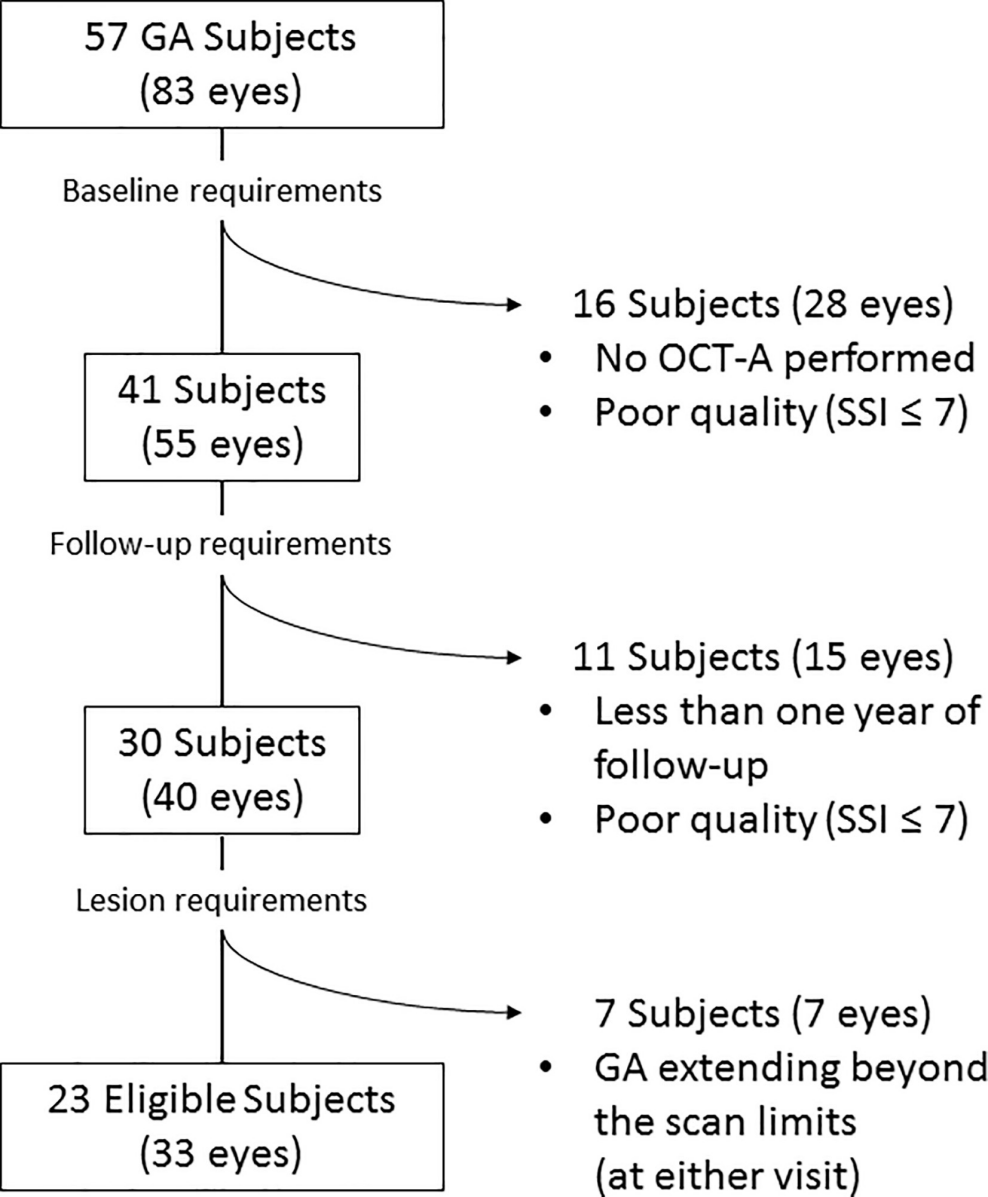
Flow chart diagram explaining the selection process of eligible eyes for the study. Among the initial cohort of 57 subjects with geographic atrophy (GA) in at least one eye, only 23 (33 eyes) met all the inclusion criteria and were included in the analysis. OCT-A: Optical coherence tomography angiography; SSI: Signal Strength Index.

Thirty-three eyes of 23 patients (5 males, mean age: 84.67 ± 6.42 years) met eligibility criteria for this retrospective analysis (Fig 3).

The mean follow-up time was 1.31 ± 0.2 years.

The mean area of the GA lesion at baseline was 2.98 ± 4.12 mm^2^ (median 0.8 mm^2^; range: 0.11–14.61 mm^2^) and at follow-up was 4 ± 4.68 mm^2^ (median 1.63 mm^2^; range: 0.19–15.61 mm^2^).

After the square root transformation the mean yearly growth rate (yGR) was 0.23 ± 0.17 mm/years.

The mean distance from the fovea was 0.86 ± 0.54 mm.

At baseline, the mean FV_OUT_ was 41.86 ± 2.71%, while FV_500_ and FV_1000_ were 46.4 ± 4.17% and 42.51 ± 2.65%, respectively. The mean ΔFV was 3.89 ± 2.6%.

**Fig 4:**
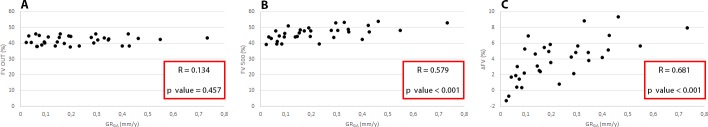
Graphic representation of the correlations explored in the study. Scatterplots showing the yearly geographic atrophy growth rate (yGR) after square root transformation and the percentage of flow voids outside the atrophy *(FV*_*OUT*_*)* (A) in the *para-atrophy region (FV*_*500*_*)* (B), and the difference between *para- and peri-atrophy regions* (ΔFV) (C). R: Pearson’s correlation coefficient; p value: two tailed p-value.

A significant correlation was found between the yGR and both flow voids in the *para-atrophy region* (FV_500_) (R = 0.579 [p<0.001]) and the ΔFV (R = 0.681 [p<0.001]), but not for FV_OUT_ (R = 0.134 [p = 0.457]) (Fig 4). No other significant correlations were found between yGR and the other parameters considered.

In the univariable analysis, the yGR was significantly associated only with the FV_500_ (regression coefficient [B] = 0.023, standard error [SE] = 0.0062, p<0.001) and with ΔFV (B = 0.044, SE = 0.0089, p<0.001).When including both variables in the multivariable analysis, only ΔFV remained significant (B = 0.038, SE = 0.012, p = 0.001) (Table 1).

**Table 1:** Univariable and multivariable generalized estimating equations analysis.

	Association with yGR			
	Univariable Analysis		Multivariable Analysis	

	Coefficient B (standard error)	p value	Coefficient B (standard error)	p value
Age	0.006 (0.0049)	0.199	NA	NA
GA area at baseline	0.003 (0.0064)	0.645	NA	NA
Distance from fovea	-0.036 (0.0511)	0.480	NA	NA
FV_500_	0.023 (0.0062)	<0.001	0.006 (0.0085)	0.510
FV_1000_	0.015 (0.0126)	0.226	NA	NA
FV_OUT_	0.007 (0.0110)	0.553	NA	NA
ΔFV	0.044 (0.0089)	<0.001	0.038 (0.012)	0.001

Results of the generalized estimating equations analysis for the association between geographic atrophy (GA) yearly growth rate (yGR) and age, GA area at baseline, distance from the fovea, percentage of flow voids (FV) in the para-atrophy region (FV_500_), FV in the peri-atrophy region (FV_1000_), FV outside the GA (FV_OUT_), and the difference between FV_500_ and FV_1000_ (ΔFV). Only the variables with significant association in the univariable analysis (FV_500_ and ΔFV) were included in the multivariable statistics.

### Repeatability assessment

The ICC of the yGR measurement between graders was 0.982 (95% confidence interval (CI) 0.964–0.991) while the distance from the fovea had an ICC of 0.997 (95%CI 0.994–0.998). The calculation of the FV_OUT_ had an ICC of 0.901 (95%CI 0.8–0.951), FV_500_ had an ICC of 0.902 (95% CI 0.863–0.959), while FV_1000_ had an ICC of 0.922 (95%CI 0.842–0.961). Finally, ΔFV had an ICC of 0.907 (95%CI 0.811–0.954).

## Discussion

In this study we retrospectively investigated the correlation between the CC flow impairment around the GA lesion and the yGR of the lesion using a commercially available SD-OCTA system.

Several studies using different approaches have demonstrated a strong association between microvascular choroidal changes and AMD in all of its stages. Histopathological studies have highlighted increasing CC alterations with age and the presence of drusen[27–29]. Interestingly, the formation of drusen may not be spatially random but may be influenced by the anatomy of the underlying CC[30,31]. More specifically for dry AMD late stages, *Biesemeier et al*. examined four post-mortem GA eyes using a combination of light and electron microscopy observing CC loss occurring in regions underlying intact retina and RPE, and concluded that CC breakdown may precede RPE degeneration in AMD[3]. Several OCTA studies have now investigated CC alterations at nearly all stages of AMD[10,12,32,33].

Recently *Moult et al*, using a swept-source (SS)-OCTA prototype, performed a qualitative analysis of the CC in eyes with nascent GA and drusen-associated GA demonstrating that the CC flow impairment is present under the area of the lesions and throughout the imaged field even in the very early stages of the disease[32].

The mechanism(s) driving the RPE alterations (i.e. drusen or atrophy) and the basis for the predilection of these alterations to form in regions associated with CC impairment, is still unknown. One hypothesis is that the CC relies on vascular endothelia growth factor (VEGF) secretion by the RPE, so the dysfunction of its cells could impair this trophic signaling process leading to endothelial cell loss[5,6]. Alternatively, primary CC vascular impairment, due to inflammatory or degenerative mechanisms or other genetic and non-genetic factors, may lead to RPE ischemia and dysfunction[3,4,34].

A recent study by our group demonstrated that Intermediate AMD eyes of patients with neovascular AMD (nAMD) in the fellow eye have an increased average CC signal void size compared to eyes without nAMD in the fellow eye. Therefore we speculated that the choroidopathy may be driving the development and progression of AMD[12].

More specifically for GA, *Sacconi et al*, using a SD-OCTA system, compared the CC flow under the regions rated as hyperautofluorescent on fundus autofluorescence (FAF) images surrounding the GA with similar regions rated as isoautofluorescent. While CC flow impairment was present in both regions, it was significantly higher in the hyperautofluorescent locations which are thought to represent regions of significant RPE impairment. Based on these findings they hypothesized that the first injury in GA occurs at the level of the CC.

Despite this mounting evidence, it is still impossible to exclude that RPE dysfunction, not revealed by current imaging modalities, may still be the primary trigger for CC flow impairment. The use of new multimodal imaging techniques including fluorescence lifetime imaging ophthalmoscopy [35,36], quantitative fundus autofluorescence [37–39], or adaptive optics imaging[40,41] may eventually provide further clarity to this issue.

**Fig 5:**
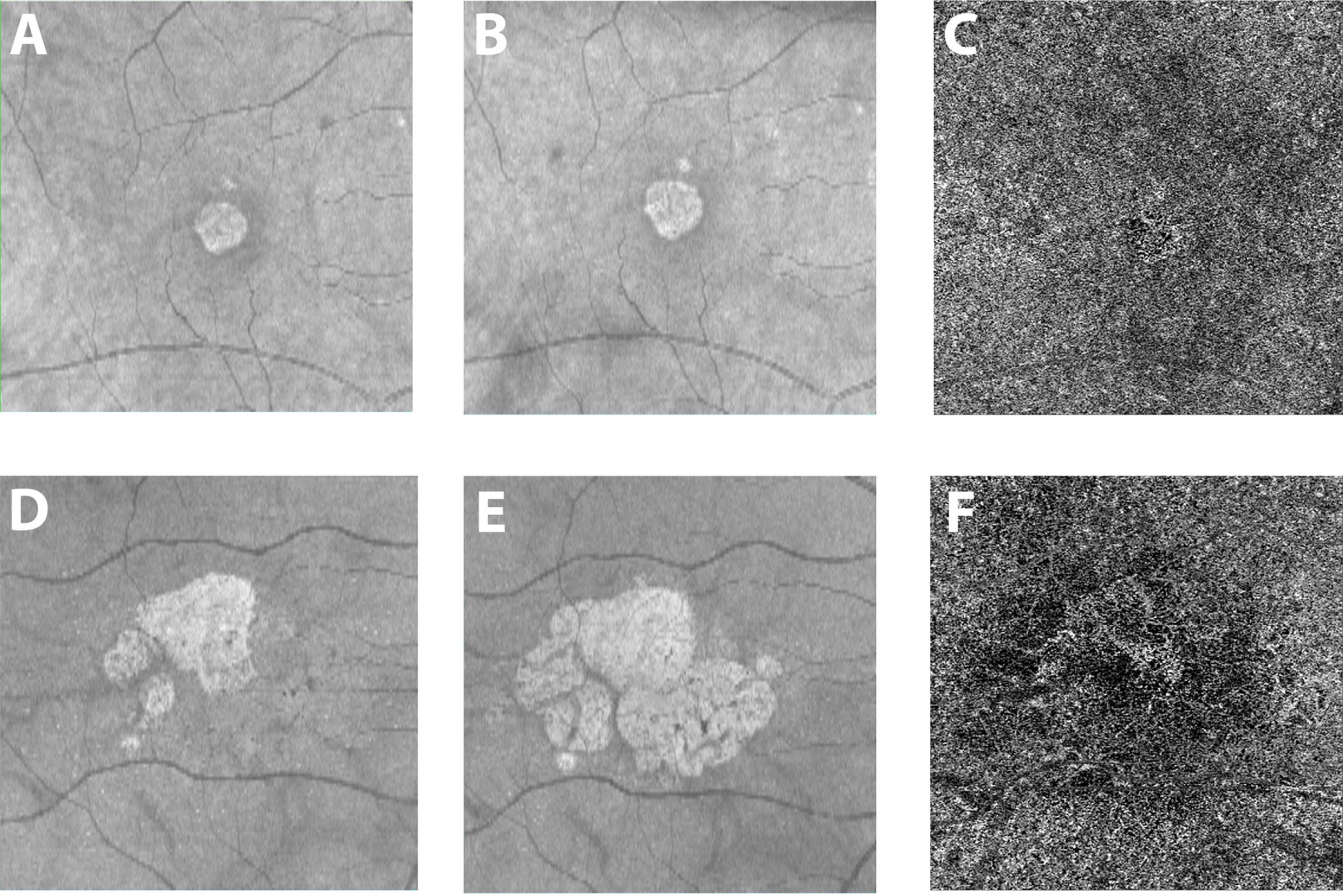
Examples of eyes with geographic atrophy and different growth rate. One eye from two patients with geographic atrophy (GA) are shown in the two rows of images. A and D are the optical coherence tomography (OCT) fundus photos at baseline while B and E were acquired after one year. The two patients show a very different yearly growth rate (0.07 and 0.73 for the first and second row respectively). The OCT angiogram at the level of the choriocapillaris (C and F) from the baseline visit for these two patients shows dramatically different flow impairment surrounding the lesion, with considerably greater flow voids in the latter case (41,2% versus 53% for the first and second row respectively).

Our study is the first to report a significant positive correlation between the CC flow impairment around the GA lesion and its growth rate independent of age (Fig 5).

However, in a recent study, we demonstrated that the distribution of the flow voids on the CC OCT angiograms of healthy eyes is not uniform, but decreases linearly with increasing distance from the fovea [42]. This phenomenon is particularly evident in older subjects (>65 years) where, in a 6x6 mm OCT-A acquisition, the physiologic reduction in the flow voids percentage is an increase of approximately 0.8% for every 500 μm away from the fovea [42]. Taking this regional effect into account, it is possible that differences in flow voids around different atrophic lesions could be confounded by differences in their dimensions or eccentricity. In order to better evaluate the impact of this possible confounder we evaluated the correlation between (1) the FV_OUT_ and (2) the ΔFV with the yGR.

**Fig 6:**
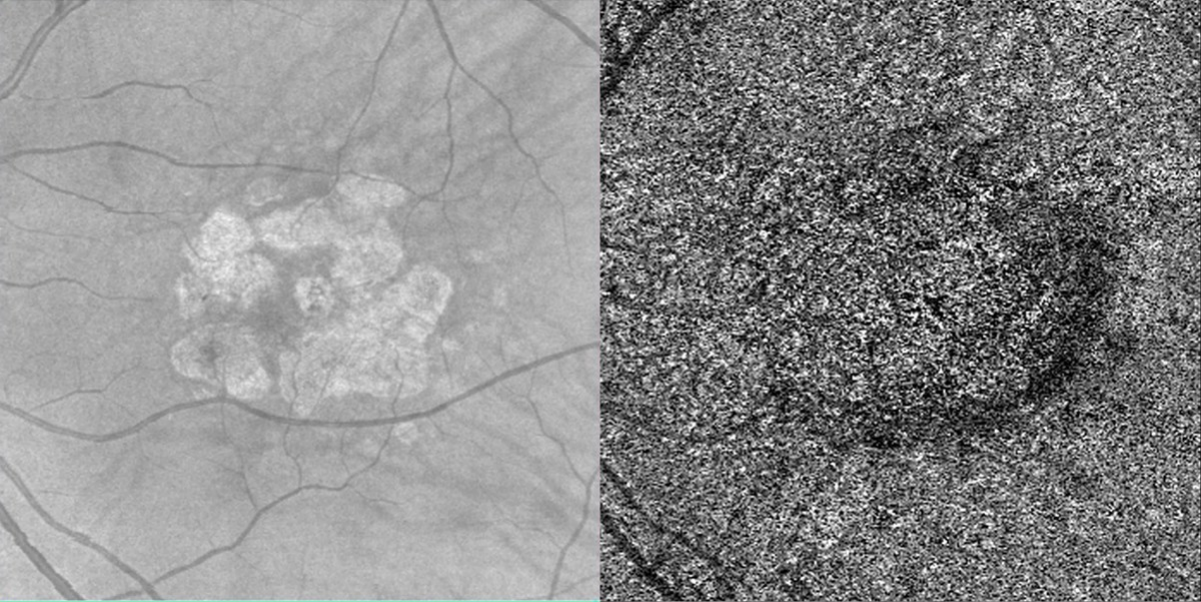
Eye with geographic atrophy demonstrating difference in flow voids between the para-atrophy region and more distant regions. One eye from a patient with geographic atrophy (GA) is shown in the two images. The optical coherence tomography (OCT) fundus photo is on the left while the corresponding OCT angiogram at the level of the choriocapillaris (CC) is on the right. In the OCT angiogram, there is a clearly evident difference in flow voids between the region outside the GA and the *para-atrophy region* (38,23% vs 47,05% respectively).

The percentage of flow voids in the entire image outside of the atrophy (FV_OUT_) should be less affected by the topographic distribution of the flow voids. Of note, the FV_OUT_ did not show a significant correlation with GA growth rate, likely because relatively preserved CC far beyond the atrophy border masked more severe areas of impairment immediately adjacent to the GA (Fig 6).

In contrast, the ΔFV between the *para- and peri- atrophy regions*, showed a significant correlation with the GA growth rate. Furthermore, when included in the same model with FV_500_, ΔFV remained strongly significant, probably because ΔFV partially contains the same information as FV_500_ but with less confounding effects. Of note these two variables are highly correlated (R = 0,79, p<0,001).

The mean ΔFV in the study was 3.89 ± 2.6%, which is higher than the expected difference of 0.8% based on regional differences alone[42]. This argues that the CC impairment in the *para-atrophy area* is a real pathological change, and not the result of the physiologic distribution of flow voids on the images. While this addresses lesions that involve the fovea (26 out of the 33 eyes in the present study), eccentrically positioned lesions may still be confounded by regional differences in the CC. In order to partially address this issue, we included the dimension of the GA and the mean distance from the fovea in our statistical model. Of note, both parameters were not associated with the growth rate. However, given the substantial variability in the shape and position of GA lesions, it may still be impossible to completely eliminate the confounding impact of regional variability in CC flow voids. Further larger studies comparing lesions of similar size, morphology, and position may more completely address this issue.

Baseline GA size was also not a predictor of growth rate in our analysis based on square root-transformed areas, confirming the effectiveness of the square root transformation in eliminating the dependence on baseline lesion size[18].

Given the absence of a significant correlation between the GA dimension at baseline and the FV we may hypothesize that CC impairment may be a key factor influencing enlargement of the GA lesion. This observation is perhaps not surprising as these regions of greater CC impairment would be expected to be associated with a greater impairment of the overlying RPE. One would expect that these more impaired RPE cells would be most susceptible to early death.

Although the precise role of alterations of the CC in the pathogenesis of GA requires further investigation, our finding that CC flow impairment may influence GA progression rate has potential implications for the selection of patients for future therapeutic clinical trials. Furthermore. the results of our study may facilitate further investigations of a topographic characterization of the CC in GA patients which may allow, in a longitudinal setting, the prediction not only the rate of GA growth overall but also its preferred direction of expansion.

Our study is not without limitations, however, including its retrospective design (with potential for selection bias), the relatively small sample size, and the performance and accuracy of the projection removal algorithms which may have affected the calculation of the flow void percentage on the CC OCT angiograms. In addition, as this was an exclusively OCT-based study, we were not able correlate these CC findings on OCTA with abnormalities on other imaging modalities such as color photographs or FAF images. This will be important in future studies as FAF features associated with GA progression rate have been previously reported. In addition, our identification of atrophy was also based on OCT alone. On the other hand, though FAF is still considered the gold standard technology to identify the areas of atrophy, *en-face* OCT has been proven to be a valuable tool in detecting these lesions (even for nascent GA)[13,24,43] and in following their progression[14]. In addition, OCT is more comfortable for patients and is more commonly obtained, and thus the identification of OCT and OCT-A based progression factors may be of value for clinical practice and for future clinical trials. Another limitation of our study is the use of an SD-OCT system for OCT angiography. Current commercially available SD-OCT machines, use a shorter wavelength (i.e. ~840 nm) and have more sensitivity loss with depth compared with swept source systems^22,23^, and thus may have more difficulty achieving adequate signal levels at the CC because of its location beneath the highly scattering RPE. The presence of drusen further exacerbates the problem. However, to mitigate this issue, we did employ a previously described signal loss compensation strategy[20]. In addition, as SD-OCT systems are still more prevalent than swept source OCT devices in clinical practice, we would argue that the approach utilized in our study is of greater clinical relevance.

Even though ΔFV seems to be the variable with the strongest association with GA yearly growth rate, FV_500_ could still be a good shortcut for the clinical evaluation of GA patients since it’s easier to compute and to evaluate (even just qualitatively).

In summary, we report a correlation between the CC flow impairment surrounding GA lesions and the growth rate of the lesions. If replicated in future prospective, longitudinal studies, measuring the FV of the CC in the *para- and periatrophy* regions may prove to be useful parameters for evaluating the prognosis of these eyes.

## Supporting information

S1 Table Database used in the study. (XLSX)
